# Familial and Bullying Victimisation: The Impact of Early Adversity Within the Home and Peer Settings on Late Adolescence and Adult Psychopathology

**DOI:** 10.1007/s40653-022-00481-2

**Published:** 2022-09-16

**Authors:** N.I. Bond, M. McLafferty, C. Lapsley, E. Ennis, E. Murray, D. Heenan, S.M. O’Neill

**Affiliations:** 1 School of Psychology, Ulster University, Coleraine, Northern Ireland; 2 Centre for Personalised Medicine, Ulster University, Derry/Londonderry, Northern Ireland; 3 School of Applied Social and Policy Sciences, Ulster University, Jordanstown, Northern Ireland

**Keywords:** ACEs, Bullying, Psychopathology, Education Policy, Latent Profile Analysis

## Abstract

**Background:**

Awareness of adverse childhood experiences and their impact on adult psychopathology primarily focuses on adversities within the home. There is limited insight into the impact of adversities across peer environments.

**Objective:**

This study investigates 19 items related to adverse experiences across the home, school and peer environments and their relationship to 12-month and lifetime psychopathology.

**Data:**

Secondary analysis of the Ulster University Student Well-being Study. The dataset included completed responses across all selected variables for 729 participants.

**Method and Results:**

Latent profile analysis identified a low adversity profile, bullying adversity profile and higher prevalence adversity profile. Regression analysis of the three profiles and demographics variables indicated their impact on adult psychopathology lifetime and 12-month prevalence rates.

**Conclusion:**

Schools and HE institutions should acknowledge the impact of childhood adversities. In doing so, it is important to consider the deeper impact of bullying due to its links with psychopathology across the lifespan. Educational institutions should take appropriate steps to mitigate continued exposure as students’ progress through the education system.

## Introduction

Since the publication of the Adverse Childhood Experience Study (Felitti et al., 1998), there is a growing body of evidence that ACEs are inter-correlated with psychiatric morbidities across the lifespan and that this effect is cumulative (Copeland et al., 2007; Bussemakers et al., 2019). Particularly, adversities stemming from maladaptive family functioning link to the development of enduring mental health problems (McLaughlin et al., 2010). Although ACEs literature generally focuses on adversities experienced within the home environment, there is a body of evidence that categorises bullying victimisation as childhood adversity, one which has a lasting impact on psychopathology (Arseneault, 2018; Finkelhor et al., 2007b; Radford et al., 2013). Adults exposed to bullying behaviour in childhood show elevated levels of anxiety, depression (Fisher et al., 2012) and increased rates of self-harm and suicide behaviours (Lereya et al., 2013). A longitudinal study conducted in the UK found that adult psychopathology outcomes were similar for those who experienced adversity within the home and those who were bullied (Takizawa et al., 2014). Research indicates that negative outcomes linked to bullying increase when victims also experience maltreatment at home (Herba et al., 2008; Fisher et al., 2012) or when exposed to multiple forms of victimisation across environments (Rijlaarsdam, Cecil, Marieke Buil, van Lier, & Barker, 2021). This is unsurprising when bullying victimisation is categorised as an adverse experience as current literature indicates the cumulative impact of multiple adversities on lifetime health outcomes. Hughes et al., (2017) summarise key literature in a systematic review noting this cumulative effect.

Research has shown an increased prevalence of psychopathology, particularly the rise in suicide ideation, anxiety disorders, panic disorders, self-harm, and depression among university students (Eisenberg et al., 2012). Within NI, students commencing Ulster University in 2015 reported a high prevalence of mental health and substance disorders (McLafferty et al., 2017). Previous publications highlighted the link between childhood adversity and adult psychopathology within NI’s general and student populations (McLafferty et al., 2015; Lagdon et al., 2021). Consistent with existing research on the topic, these findings focused on adverse experiences related to the familial environment. Yet, a recent NI based study calls for the expansion of the ACE checklist to include stressful events in other settings (MacLochlainn et al., 2021).

In NI, almost half of young people (47.5%) have experienced at least one ACE, and young people from the least deprived areas are more likely to experience no ACEs (Bunting et al., 2020). Bullying is a prevalent issue within NI schools which “impacts pupils social, emotional, psychological and educational development” (Collins et al., 2010). The most recent prevalence figures for NI, completed in 2011, highlights the prevalence of bullying within NI schools, detailing that 29% of year nine pupils were bullied in the previous two months, while 21% of year nine pupils reported bullying another pupil in the previous two months (NISRA, 2011). There are notable differences among genders, with year nine girls excluded, bullied by phone or computer more than boys, and boys reported being victims of physical or verbal bullying more than girls (NISRA, 2011). A recent meta-analysis study highlights the sparsity of bullying prevalence data across Ireland and the difficulties with inferring prevalence in smaller studies (Foody et al., 2017). Recent prevalence figures show that 16.8% of 11-19-year-olds have experienced in-person bullying, and 14.9% experienced bullying online (Bunting et al., 2020).

Bullying policy has been a common practice in the UK since the early 1990s. However, it was not a legal requirement in NI primary and post-primary schools until September 2019 (Northern Ireland Assembly, 2016). Bullying exposure is pervasive across the education sector yet viewing the experience as childhood adversity can influence bullying policy development to better support students within compulsory, further, and higher education institutions. In response to health and wellbeing concerns within university populations, the World Mental Health International College Student Initiative (WMH-ICS) conducts longitudinal research to monitor student mental health and wellbeing as they progress through higher education. Site-specific surveys have demonstrated prevalence rates for participating regions, including adult psychology outcomes related to early childhood experiences (McLafferty et al., 2015; Mortier et al., 2021; Kiekens et al., 2021). The Ulster University Student Wellbeing Study (UUSWS), part of the WMH-ICS, produced this dataset for NI.

This study aimed to explore latent adversity profiles and their relationship to 12-month and lifetime prevalence rates of psychopathology. Achieved by adapting methods used by McLafferty et al., (2018), it will account for adversity within the school and peer environments in addition to maladaptive family functioning. This secondary analysis of variables within UUSWS relating to adverse experiences before the age of 18 aims to expand upon the adversity profiles noted in the primary study (O’Neill et al., 2018), creating a more robust indicator of lifetime and 12-month psychopathology prevalence among the university student population.

## Method

In 2015, Ulster University conducted the first wave of the University Student Wellbeing Study (UUSWS). The UUSWS produced baseline prevalence estimates for lifetime and 12-month mental health disorders among students commencing UU in 2015. Analysis of this data demonstrated the link between childhood adversity profiles and adult psychopathology within this population (O’Neill et al., 2018).

The UUSWS collected data on school and peer environments, including school before the age of 18. The survey included questions regarding physical, verbal, exclusionary and cyberbullying in addition to partner violence or abuse. Further analysis of the psychopathology among this population, considering the experience of bullying and adversities within the home, could generate a more robust set of adversity profiles.

## Design

The World Mental Health International College Student Initiative (WMH-ICS) is conducting longitudinal research to monitor student mental health and wellbeing as they progress through higher education (WHO, 2015). McLafferty et al., (2017) found high rates of lifetime mood disorder, generalised anxiety disorder and suicidal behaviour, including planning, attempts and ideation. Lifetime prevalence estimates across all disorders among universities who completed the WMH-ICS ranged from 19% (Belgium) to 43% (Australia) (Auerbach et al., 2018). It found similarly high prevalence rates for 12 months with an age of onset for most disorders between ages 14–17 (Auerbach et al., 2018).

The UUSWS forms part of this WMH-ICS initiative, and its first wave of data collection commenced in September 2015 across all four Ulster University (UU) campuses. Students registered for first-year undergraduate courses who resided in the UK or ROI were eligible to participate. Students received an email a week before registration with the study information and invitation to participate. On the registration day, researchers on each campus managed recruitment; they provided participants with a unique ID card and a link to the online survey designed using Qualtrics software once they had provided written consent to participate in the study. Ulster University Research Ethics Committee granted ethical approval (REC/15/0004), and further detail of the procedures used in the primary data collection can be found in McLafferty et al., (2017) and O’Neill et al., (2018).

## Sample

The dataset included completed responses across all selected variables for 729 participants. The gender distribution for the 729 participant students includes (female, n = 456; male, n = 268; MtF transgender, n = 1; FtM transgender, n = 1). This study reports gender distribution as (female, n = 457 and Male, n = 269). The mean age of participants was 20.98years (SD = 5.56), ranging from 18 to 49., The current study categorised age groups as follows: 21 and over, n = 175; under 21, n = 551. Age grouping where chosen to reflect the median age of 21, and to be consistent with other WMH-ICS initiative studies. Overall, 661 participants identified as heterosexual, while 65 participants identified as being non-heterosexual.

## Diagnostic Assessment for Psychopathology

The UUSWS conducted as part of the WMH-ICS includes a questionnaire adapted from the WMH CIDI version 3.0 (Kessler & Ustun, 2004). This questionnaire explores the prevalence of mental health problems in accordance with ICD-11 and DSM-IV criteria. The UUSWS screens for depression, anxiety disorders, bipolar disorder, and other serious emotional problems using this instrument. WMH-CIDI has good concordance with clinical assessments (Haro et al., 2006). This secondary data analysis focuses on the prevalence of lifetime and 12-month generalised anxiety disorders (GAD) and major depressive episodes (MDE) found in the participating student population. It also considers suicide behaviours and non-suicidal self-injury responses using the self-injurious thoughts and behaviour interview (SITBI) (Nock et al., 2007). The interview items assess suicidal thoughts, planning, and attempts, across participants lifetime and within the 12months before survey completion. The SITBI has good psychometric properties for validity and reliability (Nock et al., 2007). Students were asked, “did you ever do something to hurt yourself on purpose, without wanting to die (e.g. cutting, hitting, or burning yourself)” to assess self-injury using the same instrument. Self-harm is reported alongside lifetime prevalence if participants selected the response option ‘yes’.

## Adversity Items

The early childhood section of the UUSWS asked participants to reflect on their life experiences below the age of 18. Respondents indicated using a 5-point Likert scale (very often, often, sometimes, rarely, or never), how often they experienced the events stated during that time. This secondary analysis was concerned with the experience of early adversities and their impact on psychopathology. The WHO defines adolescence as the transitional period into adulthood from childhood that spans from age ten to nineteen (WHO, 2021). However, some neuroscientists argue that changes in brain development and the critical period of neural plasticity present in adolescence in humans can span ages twelve to twenty-five (Crews et al., 2007). The sample in this study reports the prevalence of psychopathology in legal adulthood (over 18 years) which incorporates the period of late adolescence.

The researcher identified 19 items with the UUSWS measuring adversity in childhood, thirteen items associated with maladaptive family functioning and six items linked to bullying. The 19 – items selected are viewable in Table 1. The analysis reverse scored the 19- items so that higher scores in each item represented elevated levels of adverse experience.

Latent Profile Analysis (LPA) assessed the co-occurrence of 19 adverse experiences within the familial and peer environments. Those considered were grouped within the UUSWS as parental maladjustment, maltreatment by a parent/ primary caregiver, maltreatment from peers and maltreatment from a romantic partner. Adverse experiences linked to parental maladjustment include serious parental mental health problems, parental substance misuse, suicidal behaviours, involvement in criminal activity, or domestic violence. Adverse experiences related to parental maltreatment include being physically punished, physically abused, verbally insulted, emotionally abused, inappropriately touched, sexually abused, neglected, or made to complete chores too hard or dangerous for your age. Adverse experiences linked to maltreatment from peer groups include physical bullying, verbal bullying, purposeful exclusion, or cyberbullying. Negative experiences related to maltreatment from a romantic partner include partner physical violence or partner emotional abuse.

## Maladaptive Family Functioning

The 13 items measuring maladaptive family functioning derived from multiple measures. Parental Questions developed from the Army Study to Assess Risk and Resilience in Service members (Ursano et al., 2014) measured emotional, mental health and substance disorders. An adapted version of the WMH-CIDI (Kessler & Ustun, 2004) measured parental suicidal behaviour and criminal activity in addition to childhood neglect and hard chores. Adapted questions from the Adverse Childhood Experiences scale (Felitti et al., 1998) measured domestic violence, physical punishment and physical abuse, verbal insults, emotional abuse, inappropriate touching, and sexual abuse.

A paper authored by the UUSWS team included these 13-items as the measure for childhood adversity when considering sociodemographic, mental health and childhood adversity risk factors for self-harm and suicidal behaviour in College students in Northern Ireland (Author et al., 2018). This study identified an additional six adversity items. This study labels the 13-items measuring maladaptive family functioning as adversity within the familial environment.

## Bullying

Six items focused on bullying experiences before 18 years old, in the school and peer environment. These items follow the 5-point Likert scale used for the adverse childhood experiences questions, are reversed coded and identified as negative experiences within the peer environment. Items assessing physical, verbal, and exclusionary bullying were adapted from the bullying survey (Swearer & Cary, 2003). Items referring to cyberbullying and verbal/physically incidents with a partner were adapted from the WMH-CIDI (Kessler & Ustun, 2004).

## Data Analysis

The Latent Profile Analysis (LPA) conducted in this study follows the process outlined in O’Neil et al. 2018. It differs only by including 19-items childhood adversity items. Table 1 presents endorsement rates for each of the 19 adverse childhood experience items, including those in the familial and peer environment. Applying weights created for the UUSWS using the gender and age characteristics of the first-year student population at Ulster University ensured the percentage reported in Table 1 is representative of the UU student population. A range of fit indices compared adversity profile models identified using latent profile analysis (LPA). Lower AIC, BIC and SSABIC and an entropy value closest to 1 identified the optimal number of profiles. Logistic regression analyses identified risk factors for lifetime and 12-month major depressive episodes (MDE), generalised anxiety disorder (GAD), self-harm, suicidal ideation, planning, and attempts. Class membership within the retained profile model were dummy coded by the researcher into a categorical variable entered the regression analysis as a covariate alongside the sociodemographic variables. Analyses were conducted using SPSS (version 24) and Mplus version 8.0 (Muthen & Muthen, 1998).

## Results

### Prevalence of Childhood Adversity Items

Endorsement rates across the 19 adverse childhood experience items varied. The highest endorsement rates were for parental mental health, parental substance misuse, receiving insults, and emotional abuse within the familial environment. Within the peer environment, physical, verbal, and exclusionary bullying had the highest endorsement rates. The cumulative weighted values for having experienced an adversity item, often or very often, shows 12.7% of females had a parent with a serious mental health problem compared to males (7.5%). Both genders report similar levels of experiencing parental substance misuse, males (6.4%), females (7.3%), and experience of repeated insults, males (6.1%), females (7.1%). Females reported a higher endorsement of experiencing emotional abuse (4.8%) than males (2.9%). The highest endorsement value for both genders was verbal bullying; 19.7% of females experienced verbal bullying very often or often, and 20.7% of males. Exclusionary bullying was frequently experienced by 20.5% of females compared to 10.3% of males.

**Table 1:** Endorsement rates for childhood adversity items

Item questions	*Very Often*, *n*, *%*		*Often*, *n*, *%*		*Sometimes*, *n*, *%*		*Rarely*, *n*, *%*		*Never*, *n*, *%*	
	*Male*	*Female*	*Male*	*Female*	*Male*	*Female*	*Male*	*Female*	*Male*	*Female*
*One of your parents had a serious mental health problem.*	*11* *3.3%*	*30* *6.5%*	*12* *4.2%*	*29* *6.2%*	*17* *6.2%*	*45* *9.9%*	*31* *11.3%*	*62* *13.6%*	*200* *75%*	*291* *63.6%*
*One of your parents had a serious alcohol or drug problem.*	*7* *2.1%*	*19* *4.1%*	*12* *4.3%*	*15* *3.2%*	*10* *3.5%*	*22* *4.7%*	*14* *5.1%*	*16* *3.5%*	*228* *85%*	*385* *84.3%*
*One of your parents attempted or died by suicide.*	*0* *-*	*7* *1.5%*	*0* *-*	*5* *1.1%*	*3* *1.1%*	*11* *2.3%*	*6* *2.1%*	*11* *2.4%*	*262* *96.8%*	*423* *92.5%*
*One of your parents was involved in criminal activities.*	*0* *-*	*2* *0.4%*	*1* *0.4%*	*5* *1.0%*	*7* *2.6%*	*7* *1.5%*	*2* *0.7%*	*11* *2.4%*	*261* *96.3%*	*432* *94.4%*
*Your parents hit or were violent to each other.*	*3* *1.0%*	*5* *1.1%*	*2* *0.7%*	*11* *2.4%*	*14* *4.6%*	*18* *3.8%*	*10* *3.7%*	*32* *7.0%*	*242* *90.0%*	*391* *85.5%*
*Someone in your family hit you so hard it left bruises or marks.*	*3* *0.9%*	*5* *1.1%*	*2* *0.7%*	*5* *1.1%*	*13* *4.8%*	*20* *4.3%*	*26* *9.4%*	*45* *9.7%*	*227* *84.2%*	*382* *83.6%*
*You were physically abused at home.*	*2* *0.6%*	*4* *0.9%*	*1* *0.4%*	*7* *1.5%*	*6* *2.0%*	*7* *1.5%*	*7* *2.7%*	*15* *3.2%*	*255* *94.3%*	*423* *92.5%*
*Someone in your family repeatedly said hurtful or insulting things to you.*	*7* *2.4%*	*15* *3.2%*	*10* *3.7%*	*18* *3.9%*	*20* *7.0%*	*48* *10.3%*	*29* *10.3%*	*54* *12.0%*	*205* *76.5%*	*322* *70.4%*
*You were emotionally abused at home.*	*5* *1.7%*	*11* *2.4%*	*4* *1.2%*	*11* *2.4%*	*8* *2.9%*	*22* *4.8%*	*15* *5.4%*	*31* *6.7%*	*239* *88.8%*	*382* *83.5%*
*Someone in your family touched you or made you touch them in a sexual way against your will.*	*0* *-*	*3* *0.6%*	*0* *-*	*1* *0.2%*	*1* *0.4%*	*4* *0.9%*	*1* *0.4%*	*3* *0.6%*	*269* *99.2%*	*446* *97.4%*
*You were sexually abused at home.*	*0* *-*	*2* *0.4%*	*0* *-*	*1* *0.2%*	*0* *-*	*5* *1.1%*	*1* *0.4%*	*6* *1.3%*	*270* *99.6%*	*443* *96.8%*
*You were seriously neglected at home.*	*1* *0.3%*	*2* *0.4%*	*1* *0.4%*	*4* *0.9%*	*2* *0.6%*	*7* *1.5%*	*7* *2.5%*	*10* *2.1%*	*260* *96.3%*	*434* *94.9%*
*You had to do chores too hard or dangerous for someone your age.*	*0* *-*	*2* *0.4%*	*2* *0.7%*	*3* *0.6%*	*3* *1.0%*	*7* *1.5%*	*16* *5.7%*	*15* *3.2%*	*250* *92.6%*	*430* *94.0%*
*How often were you bullied at school physically? (i.e., repeatedly punched, shoved, or physically hurt)*	*9* *3.1%*	*7* *1.5%*	*7* *2.5%*	*12* *2.6%*	*28* *10.1%*	*17* *3.7%*	*68* *24.5%*	*61* *13.2%*	*159* *59.8%*	*361* *79.1%*
*How often were you bullied at school verbally? (i.e., teased or called names)*	*20* *7.2%*	*29* *6.2%*	*29* *10.8%*	*62* *13.5%*	*67* *24.1%*	*103* *22.6%*	*76* *28.3%*	*113* *24.8%*	*79* *29.6%*	*151* *33.0%*
*How often were you bullied at school by someone who purposefully ignored you, excluded you, or spread rumours about you?*	*11* *3.8%*	*35* *7.6%*	*18* *6.5%*	*59* *12.9%*	*41* *15.2%*	*100* *21.8%*	*59* *21.6%*	*109* *24.0%*	*142* *52.9%*	*155* *33.7%*
*How often were you bullied over the internet (e.g., Facebook, twitter) or by text messaging?*	*3* *1.0%*	*8* *1.7%*	*2* *0.7%*	*16* *3.5%*	*17* *6.6%*	*55* *12.1%*	*45* *17.2%*	*90* *19.7%*	*204* *74.4%*	*289* *63.0%*
*How often where you in a romantic relationship were your partner repeatedly hit or hurt you?*	*0* *-*	*7* *1.5%*	*1* *0.4%*	*9* *2.0%*	*3* *1.1%*	*5* *1.1%*	*6* *1.9%*	*20* *4.3%*	*621* *96.6%*	*417* *91.1%*
*How often were you in a romantic relationship where your partner repeatedly said hurtful or insulting things to you?*	*2* *0.8%*	*8* *1.7%*	*5* *1.8%*	*19* *4.1%*	*7* *2.3%*	*30* *6.5%*	*25* *8.6%*	*44* *9.6%*	*232* *86.6%*	*357* *78.1%*

Note: n = unweighted number of participants, % = weighted

### LPA of Early Adversity at Home and in Peer Environments

LPA assessed the co-occurrence of 19 adverse experiences within the familial and peer environments. Table 2 lists a series of LPA models produced using Mplus version 8 and fit indices. The three-class model was the most optimal, with lower AIC, BIC, and SSABIC than the two-class model. The entropy value for the three-class model was good at 0.989. The LRT value was non-significant, indicating the three-class model had greater parsimony than previous models. Within the three-class model, the third profile accounted for only 3.5% of the sample. Profiles containing less than 5% can be spurious (Marsh et al., 2004), however on reviewing the profile plots, this class had a distinct pattern that was not observable in the two-class model. The observed pattern was consistent with the three-class model retained in O’Neill et al., 2018 for the maladaptive family functioning items. The fit indices for the four and five class models were lower than that of the three-class; however, the profile membership for these models was particularly low, with one or more profiles accounting for less than 2% of the sample. No model had significant LRT suggesting parsimony in all those tested; the three-class model was retained after considering all the factors.

**Table 2:** Fit indices for Latent Profile Models of childhood adversity’s experienced in familial and peer environments

Model	Log-likelihood	AIC	BIC	SSABIC	Entropy	LRT	(p)
2	-15828.437	31848.874	32289.675	31984.845	0.998	3410.194	(0.7977)
3	**-14982.925**	**30235.851**	**30855.727**	**30427.059**	**0.989**	**1684.471**	**(0.7610)**
4	-14407.150	29162.300	29961.251	29408.746	0.989	1147.089	(0.8495)
5	-13992.959	28411.917	29389.944	28713.602	0.991	775.467	(0.7695)

Note: AIC = Akaike information criterion, BIC = Bayesian information criterion, SSABIC = sample size adjusted BIC, LRT = Lo-Mendell-Rubin adjusted likelihood ratio test. The optimal model is highlighted in bold

**Fig. 1:**
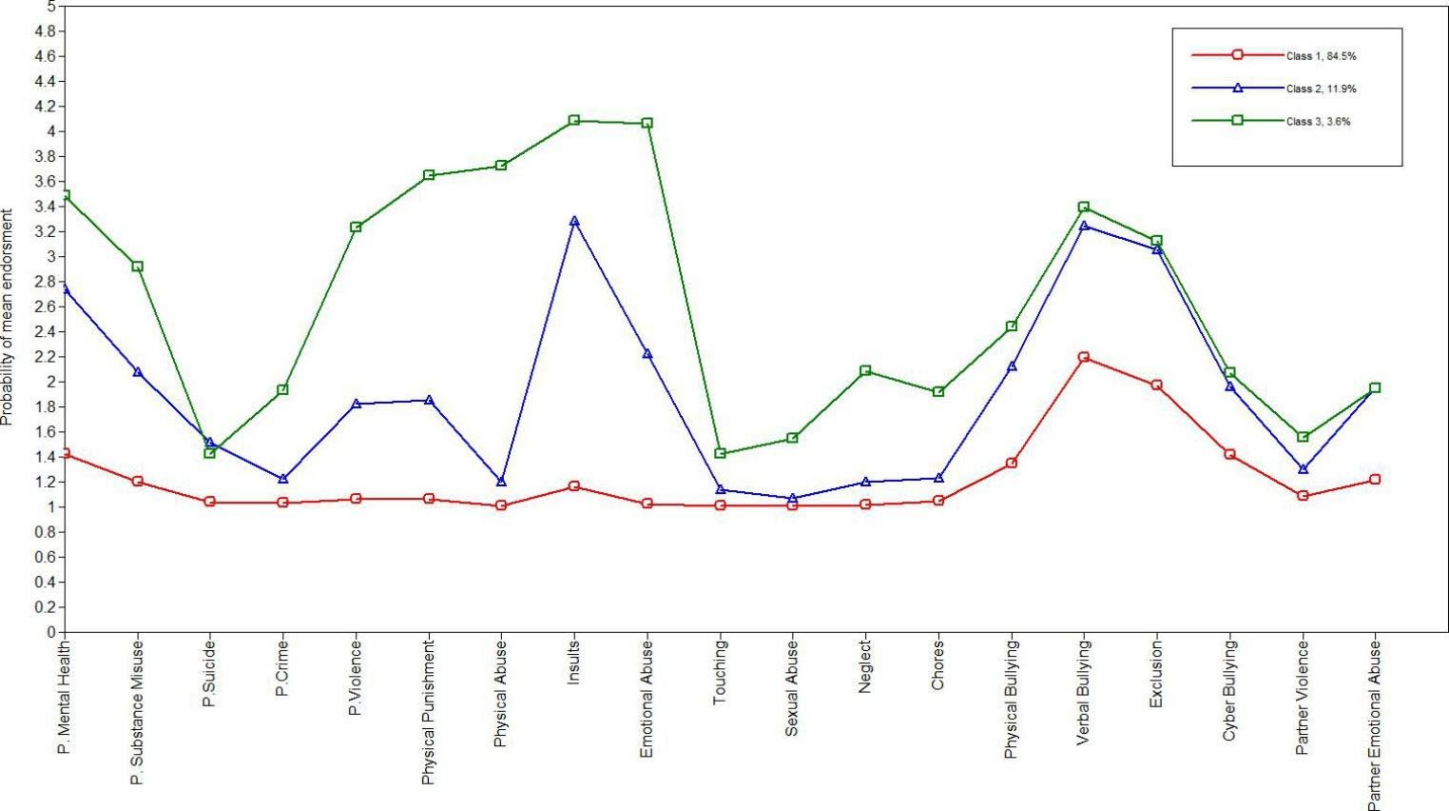
Three class model of early adverse experiences within the familial and peer environment, latent profile plot. Note: Class 1 = Baseline, Low Adversity Profile (LAP), Class 2 = Bullying Adversity Profile (BAP), Class 3 = High Prevalence Adversity Profile (HPAP)

Figure 1 illustrates the latent profile plot for the three-class model. Class, one accounts for 84.5% of the sample; it is the baseline class for subsequent analysis. It is labelled the Low adversity profile (LAP) because it is characterised by low levels of adversity within the familial environment and a moderate elevation in the negative experience of physical, verbal exclusionary and cyberbullying. Class two accounts for 11.9% of the sample; it is characterised by moderately elevated levels of parental mental health, parental substance misuse, parental violence, and physical punishment with a high increase in the experience of insults. Participants within class two also experienced moderately elevated physical and cyberbullying levels with a high elevation of verbal and exclusionary bullying within the peer environment. Class two was named the bullying adversity profile (BAP) due to the high elevation of verbal and exclusionary bullying within the peer environment and exposure to insults and emotional abuse within the familial environment. Class three accounts for 3.6% of the sample; it is characterised by elevated levels across all the adverse experiences within the home environment, except for parental suicide, and across all negative experiences within the peer environment. Parental violence, physical punishment, physical abuse, insults, and emotional abuse are the highest elevated adversities within the familial environment. The highest elevations within the peer environment are for physical, verbal, and exclusionary bullying. Class three was named the high prevalence adversity profile (HPAP) as participants consistently experienced higher occurrence levels of adversities across the familiar and peer environments within this class.

### Logistic Regression Analysis

A series of logistic regression analyses examined associations between sociodemographic variables and the three adversity profiles with lifetime mental health problems, suicidality, and self-harm. The psychopathology measures were the dependent variables, sociodemographic variables entered as covariates, and the adversity profiles retained within a categorical variable were entered as covariates. Further Logistic regression analyses were completed for 12-month mental health problems and suicidality indicators.

### Lifetime Prevalence Rates

Table 3 presents the results following the regression analysis completed for sociodemographic variables and adversity profiles with lifetime mental health prevalence rates. Consistent with findings from (O’Neill et al., 2018), female students were more likely to have MDE (*OR* = 1.530, p < .05), GAD (*OR* = 1.556, p < .05) and were more likely to self-harm (*OR* = 2.284, p < .001) compared to males. No significant gender difference was found for lifetime prevalence of suicide attempts or planning, although females were more likely than males to endorse lifetime suicide ideation (*OR* = 1.710, p < .01). Compared to students under 21 years, students over 21 years old were more likely to have made a suicide attempt in their lifetime (OR = 1.914, p < .05). Yet, no significant difference was found in the prevalence of suicide ideation or planning. Additionally, this older age group has an increased likelihood of having experienced a major depressive episode (*OR* = 1.628, p < .05) but are less likely to have self-harmed (*OR* = 0.540, p < .05) in their lifetime.

Students who identified as non-heterosexual make up 8.9% of the student population at UU. They were consistently more likely to have experienced a range of problems in their lifetime when compared to heterosexual students. Non-heterosexual students were more than three times more likely to develop GAD (*OR* = 3.341, p < .001) and almost three times more likely to have experienced an MDE (*OR* = 2.748, p < .001) than their heterosexual peers. Starkly, in contrast to the heterosexual student population, non-heterosexual students were over five times as likely to have self-harmed (*OR* = 5.109, p < .001), had thoughts of suicide (*OR* = 5.187, p < .001), made a suicide plan (*OR* = 5.627, p < .001) and had made at least one suicide attempt (*OR* = 5.283, p < .001) in their lifetime.

BAP and HPAP students, at minimum levels, are over twice as likely to have experienced lifetime MDE and GAD, have a history of self-harm, suicide ideation, and made both a suicide plan and attempt in their lifetime when compared to LAP students. HPAP students experienced the highest endorsement rates of adverse experience within the familial environment and negative experiences with the peer environment, which included all forms of bullying noted within the survey. This group was over four times as likely as the LAP to have self-harmed (*OR* = 4.806, p < .001), experienced suicide ideation (*OR* = 4.909, p < .001) and developed GAD (*OR* = 4.605, p < .001) in their lifetime. Concurrently, HPAP students were over six times more likely to have made a suicide attempt (*OR* = 6.598, p < .001) or plan (*OR* = 6.409, p < .001). BAP students experienced fewer adversities within the familial environment than HPAP students yet had similar bullying experiences within the peer environment, albeit endorsed at a lower rate. Unsurprisingly BAP students where consistently more likely than LAP students to develop mental health problems; GAD (*OR* = 3.692, p < .001), MDE (*OR* = 4.011, p < .001), have self-harmed (*OR* = 4.332, p < .001), or engaged in suicidal behaviours across their lifespan; ideation (*OR* = 5.208, p < .001), attempt (*OR* = 3.195, p < .01), plan (*OR* = 5.133, p < .001). Conversely, BAP students were more likely than HPAP students to have developed MDE and experienced thoughts of suicide in their lifetime.

**Table 3:** Logistic regression analyses of demographic variables and adverse childhood experiences within the familial and peer environment correlate with mental health problems and suicidal behaviour within participants’ lifetime

Demographics N = 729		Self-Harm (n = 150) OR (95% CI)	MDE (n = 185) OR (95% CI)	GAD (n = 172) OR (95% CI)	Suicide ideation (n = 234) OR (95% CI)	Suicide attempt (n = 59) OR (95% CI)	Suicide planning (n = 147) OR (95% CI)
**Gender** Female Male	(457) (269)	2.284*** (1.5–3.5) 1.0	1.530* (1.1–2.2) 1.0	1.556* (1.1–2.3) 1.0	1.710** (1.2–2.4) 1.0	1.495 (0.8–2.8) 1.0	1.301 (0.9-2.0) 1.0
**Age** 21 and over Under 21	(175) (551)	0.540* (0.3–0.9) 1.0	1.628* (1.1–2.5) 1.0	1.152 (0.7–1.8) 1.0	1.180 (0.8–1.8) 1.0	1.914* (1.0-3.6) 1.0	1.348 (0.8–2.2) 1.0
**Sexuality** Non-heterosexual Heterosexual	(65) (661)	5.109*** (2.9-9.0) 1.0	2.748*** (1.6–4.8) 1.0	3.341*** (2.0-5.8) 1.0	5.187*** (2.9–9.2) 1.0	5.283*** (2.7–10.5) 1.0	5.627*** (3.2–9.9) 1.0
**Adversity profile** HPAP BAP LAP	(28) (91) (607)	4.806*** (2.0-11.7) 4.332*** (2.6–7.3) 1.0	2.697* (1.2–6.2) 4.011*** (2.5–6.5) 1.0	4.605*** (2.0-10.6) 3.692*** (2.3-6.0) 1.0	4.909*** (2.1–11.7) 5.208*** (3.2–8.6) 1.0	6.598*** (2.6–17.1) 3.195** (1.6–6.4) 1.0	6.409*** (2.7–15.0) 5.133*** (3.1–8.5) 1.0

Note: *OR* = odds ratio, CI = confidence intervals, significance values *p < .05, **p < .01, ***p < .001

### 12-Month Prevalence

Table 4 presents regression analysis of sociodemographic variables and adversity profiles with mental health prevalence rates in the 12-months before completing the UUSWS. In contrast to the odds ratios found for the lifetime prevalence rates considering gender, being female was only significant for having experienced GAD (OR = 1.544, p < .05) and MDE (OR = 1.518, p < .05) in the past 12months. Age was not a significant predictor for suicide ideation or having made a suicide plan within the lifetime prevalence response. However, students who are over 21 were almost two thirds less likely to have experienced suicide ideation (*OR* = 0.337, p < .001) or planned (*OR* = 0.380, p < .001) in the last 12 months. Converse to the lifetime prevalence rates, being over 21 years old was not a significant predictor of having made a suicide attempt within the 12 months prior to completing the UUSWS. While not definitive, the difference noted in lifetime and 12month prevalence rates for suicide ideation, planning, and attempt, may indicate that age is a protective factor in suicidology, with students more likely to have experienced this in their younger years. Consistent with the lifetime prevalence rates, students over 21 were almost one and half times more likely to have experienced MDE (OR = 1.423, p < .05) in the last 12months. Identifying as non-heterosexual remains a significant indicator for a range of problems prevalent in the 12 months prior to completing the UUSWS. Compared to students who identified as heterosexual, non -heterosexual students are two to three times more likely to have experienced MDE (*OR* = 2.649, p < .001), GAD (*OR* = 3.546, p < .001), suicide ideation (*OR* = 2.255, p < .01) and engaged in suicide planning (*OR* = 2.330, p < .01). BAP and HPAP students had elevated likelihoods of experiencing all reported mental health problems within the past 12 months, except for having made a suicide attempt. BAP and HPAP students are at least over three times as likely as LAP students to have experienced MDE, GAD or engaged in suicidal behaviours in the last 12-months. Similar to the lifetime prevalence findings, BAP students are more likely than those with HPAP to have experience suicide ideation(*OR* = 5.230, p < .001) or MDE(*OR* = 3.529, p < .001), albeit the difference in *OR* for MDE is marginal within the 12-month prevalence compared to the variation noted for lifetime prevalence.

**Table 4:** Logistic regression analyses of demographic variables and adverse childhood experiences within the familial and peer environment correlates of mental health problems and suicidal behaviour within the past 12months

Demographics N = 729		MDE (n = 185) OR (95% CI)	GAD (n = 172) OR (95% CI)	Suicide ideation (n = 234) OR (95% CI)	Suicide attempt (n = 59) OR (95% CI)	Suicide planning (n = 147) OR (95% CI)
**Gender** Female Male	(457) (269)	1.518* (1.0-2.2) 1.0	1.544* (1.1–2.3) 1.0	1.136 (0.8–1.7) 1.0	0.761 (0.3–2.2) 1.0	0.875 (0.5–1.4) 1.0
**Age** 21 and over Under 21	(175) (551)	1.423* (0.9–2.2) 1.0	1.057 (0.7–1.7) 1.0	0.337*** (0.2–0.6) 1.0	0.659 (0.2–2.7) 1.0	0.380** (0.2–0.8) 1.0
**Sexuality** Non-heterosexual Heterosexual	(65) (661)	2.649*** (1.5–4.6) 1.0	3.546*** (2.0-6.1) 1.0	2.255** (1.3-4.0) 1.0	1.593 (0.4–6.9) 1.0	2.330** (1.2–4.5) 1.0
**Adversity profile** HPAP BAP LAP	(28) (91) (607)	3.041** (1.3–6.9) 3.529*** (2.2–5.7) 1.0	5.026*** (2.2–11.6) 3.677*** (2.2-6.0) 1.0	3.951** (1.6–9.9) 5.230*** (3.1–8.9) 1.0	2.204 (0.2–20.9) 2.120 (0.5–8.2) 1.0	5.130*** (1.9–13.7) 4.965*** (2.7-9.0) 1.0

Note: *OR* = odds ratio, CI = confidence intervals, significance values *p < .05, **p < .01, ***p < .001

## Discussion

This study expanded upon the adversity profiles found in (O’Neill et al., 2018) by considering the additional impact of adversity within the peer environment, specifically bullying. Doing so generated three new distinct adversity profiles, low adversity profile (LAP), bullying adversity profile (BAP) and the highest prevalence of adversity profile (HPAP). The prevalence of bullying item endorsement across all profiles indicated that bullying exposure is pervasive in the education system, impacting most students, not just those who experience adversities in other settings. The discussion below highlights how the inclusion of bullying alongside exposure to maltreatment at home alters late adolescent and adult psychopathology outcome likelihoods when compared to the primary study. It is hoped that the current study creates a more robust measurement of the cumulative impact of adversity exposure across environments.

Klomek et al. (2015) synthesised multiple large-scale studies conducted between 1960 and 2015 suggesting a link between exposure to bullying and lifetime psychopathology, notably depressive disorders, and suicidality. These findings are consistent with those reported in O’Neill et al., 2018 who found that high levels of adversity significantly increased the likelihood of suicide behaviours and self-harm within participants lifetime. With the addition of bullying items, this study found that HPAP students were less likely than the high-risk group in O’Neill et al., 2018, to engage in self-harm and suicide behaviours or develop MDE, or GAD. Conversely, the OR for BAP students experiencing the same psychopathologies across the lifespan and within 12-months are higher than those found for the moderate-risk group. These differences suggest that the pattern of adversity across environments can have greater impact than the prevalence level. The addition of bullying items changes the predictive power of the adversity profiles for the same sample group, which can better inform the supports offered within the university.

This study found that prevalence rates across all adversity items within BAP are lower than that of the HPAP class. Consistent with existing literature, students with the highest prevalence rate of adversities were more likely to experience GAD, self-harm, make a suicide attempt or plan. Rates are highest for the HPAP group at 12-months for GAD and suicide planning. However, students with the BAP profile are more likely to have developed MDE or had thoughts of suicide in their lifetime. This increased likelihood is present in the 12month data, although the difference for experiencing MDE between the BAP and HPAP class at this time frame is marginal. While the endorsement pattern for bullying items is similar across all profiles, albeit at different levels, there is a unique pattern within the BAP profile across all adversity items. When the prevalence of verbal and exclusionary bullying in school mirrors insults and emotional abuse within the familial environment, as in the BAP class, the cumulative impact of similar adversities across multiple environments may contribute to the increased likelihood noted. Further studies could consider if this pattern is unique to non-physical mal-treatment or if a consistent pattern for exposure to physical abuse within multiple environments would also create a distinct adversity profile.

Higher education institutions seeking to support students should improve their understanding of childhood adversities and their effects across the lifespan. This includes acknowledging that adversities experienced within the education sector contribute to that cumulative impact, such as bullying. In addition to the distinct adversity profiles, findings support those found in similar studies where females were more likely than males to have experienced MDE, GAD, self-harmed or expressed suicide ideation in their lifetime, with increased likelihood noted for MDE and GAD within the past 12-months (Said et al., 2013). Identifying as a non-heterosexual student remains a significant predictor of increased prevalence for all mental health disorders, self-harm, and suicide behaviours, except for having made a suicide attempt in the past 12 months, which is similar to previous findings using the primary data (McLafferty et al., 2017).

Interestingly students over 21 were almost twice as likely to have made a suicide attempt in their lifetime; however, this same group were nearly two thirds less likely to have experienced suicide ideation or made a suicide plan in the last 12 months. Although not statistically significant, the OR indicates over 21s are less likely to have made a suicide attempt in the past 12 months. It would suggest that engagement in suicide behaviours is more prevalent in their younger years for this participant population. However, it was unclear if this trend was noted within O’Neill et al., 2018 as 12-month suicide behaviour rates were not reported. This finding would support studies that argue that suicide attempts in adolescence are more common (Ferguson et al., 2005). However, other studies such as (Dhossche et al., 2002) or (Pfeffer et al., 1993), indicate a history of suicidality increases the risk of making future suicide attempts. Yet this study would suggest, students who have made a suicide attempt in the past are less likely to in the future. However, this sample has a higher proportion of participants under 21 at the time of completing the UUSWS. It is possible that a longitudinal study of this sample would demonstrate a different trend in suicide behaviours more indicative of the patterns noted in previous studies, indicating further research is required.

## Limitations

The UUSWS questionnaire design relies on self-report measures criticised for being unreliable, specifically when recalling historical events. However, most participants were under the age of 21, which reduced the bias in identifying events before 18. The study’s cross-sectional nature, with responses taken at a one-time point, precludes the identification of cause-and-effect relationships. Females are overrepresented within this study which may account for the increased lifetime and 12-month mental health disorder prevalence noted for sociodemographic variable gender difference. However, weights were applied to mitigate this. Overall, the findings from this study are helpful in designing supports for study participants during their time at Ulster University, yet results are not necessarily generalisable to other populations.

## Conclusion

Previous studies have highlighted an increased prevalence of mental health disorders among HE students, which these findings support. While it is difficult to determine the overall population these findings represent, the data presented indicate the demographic and adversity profile variables for students who may need additional support throughout their HE courses of study. As these rates have been determined upon entry into, HE, this research provides a retrospective insight into the early life experiences, including time in education before UU access, and therefore of interest to the HE and post-primary education sector. Providing evidence that bullying can have a long-term negative impact on psychopathology should encourage all school sectors to take anti-bullying measures. The authors recommend that any measures used be trauma informed and have a strong evidence base to support their use within an educational setting.

These findings have implications for the practice used within schools to target and resolve bullying behaviours, which in part can impact the adversity profiles students can develop across their time in education. The NI education system has limited capacity to pre-emptively control or influence the adversities experiences within the familial environment, resulting in reactionary support systems. However, it is within the education system’s remit to address awareness and control over the school environment, wherein bullying behaviour is likely experienced. There are already moves within NI to prioritise bullying prevention; as of September 2019, all schools must have an anti-bullying policy and mandatory staff training (Northern Ireland Assembly, 2016). However, the practice shift needs to include support for students who have experienced bullying alongside adversity across the home and peer environments to limit the potential lifetime impact, which is not evident within NI (NISRA, 2011). Education institutions seeking to improve trauma-informed practices cannot eradicate lifetime psychopathology linked to childhood adversity within the home. Still, they can reduce the cumulative impact of adversity across environments by mitigating bullying exposure within all educational settings.

In this context, the participants’ experiences within this study can inform intervention, prevention, and support programmes at pre-and post-higher education institutions in NI. It expands the discussion regarding childhood adversity and challenges the education sector to foster a safer learning environment.
